# Achieving ethnic equality in the Israel trauma healthcare system: the case of the elderly population

**DOI:** 10.1186/s13584-019-0294-8

**Published:** 2019-02-13

**Authors:** Nura Abdel-Rahman, Nechemia Yoffe, Maya Siman-Tov, Irina Radomislensky, Israel Trauma Group, H. Bahouth, A. Becker, A. Hadary, I. Jeroukhimov, M. Karawani, B. Kessel, Y. Klein, G. Lin, O. Merin, B. Miklush, Y. Mnouskin, A. Rivkind, G. Shaked, G. Sibak, D. Soffer, M. Stein, M. Wais, H. Pharan, I. Garbetzev, Kobi Peleg

**Affiliations:** 1 Israel National Center for Trauma and Emergency Medicine, The Gertner Institute for Epidemiology and Public Health Policy, Tel- Hashomer, 52621 Ramat Gan, Israel; 2 Department of Disaster Management, School of Public Health, Tel Aviv University, Tel Aviv, Israel

**Keywords:** Injury, Seniors, Minority, Hospitalization, Rehabilitation

## Abstract

**Objective:**

To determine if ethnic disparities exist with regard to the risk of injury and injury outcomes among elderly hospitalized casualties in Israel**.**

**Methods:**

A retrospective study based on data from the Israeli National Trauma Registry between 2008 and 2017. Data included demographic, injury and hospitalization characteristics. Descriptive statistics and adjusted logistic regression were used to examine the differences between Jewish and Arab casualties, aged 65 and older.

**Result:**

The study included 96,795 casualties. The proportion of elderly hospitalized casualties was 2.8 times greater than their proportion in the population (3.1 times greater among Jews and 2.1 times among Arabs). In comparison to Arabs, Jews suffered from a greater percentage of head injuries (10.5 and 8.9%, respectively for Jews and Arabs *p* < .001), but fewer extremity injuries (46.7% vs. 48.0% respectively for Jews and Arabs *p* < .05). Among severe/critical casualties and among casualties with severe head injuries, Arabs were more likely to be transported to the hospital in a private car (27% vs. 21% respectively for Arabs and Jews p < .001; 30.5% vs. 23.3% respectively for Arabs and Jews p < .001). Logistic regression analysis, adjusted for age, gender, injury severity, type of injury, type of trauma center and year of admission, shows that Jews, relative to Arabs, were more likely to be hospitalized for more than seven days, admitted to the intensive care unit (ICU) and to be discharged to a rehabilitation center (OR: 1.3, 1.3 and 2.4 respectively). No differences regarding surgery (OR: 0.95) or in-hospital mortality (OR: 0.99) were found.

**Conclusions:**

Ethnic disparities between Jewish and Arab hospitalized casualties were observed with regard to hospital stay, ICU admission and rehabilitation transfer. However, no differences were found with regard to mortality and surgery. While the reported disparities may be due in part by cultural differences and accessibility, health policy decision makers should aim to reduce the gaps by optimizing the accessibility of ambulance and rehabilitation services as well as increasing awareness regarding the availability of these medical services among the Arab population.

## Introduction

The elderly population continues to expand, resulting in a greater number of geriatric patients being at risk for injury [1]. The increase of traumatic events involving the geriatric population is due to longer life expectancy, increasing activity and mobility, and a progressive improvement in quality of life [2]. In comparison to younger patients, outcomes following injury are significantly worse among the geriatric population, resulting in disproportionate healthcare utilization and expenditures. Disparities in the risk of injury and health outcomes between ethnic groups have been demonstrated in a number of countries around the world. Minority ethnic groups are often at higher risk than the majority of the population [3–8]. A recent study from the United States has demonstrated that trauma casualties among minority populations are clustered in hospitals with higher than expected mortality [9]. While injury research studying ethnic disparities has focused on the younger population, research of ethnic disparities among the elderly is lacking [10]. In addition, research among the non-American trauma patient population has been limited [11].

The majority of the elderly population in Israel is Jewish (79.3%) with an Arab minority (20.7%). Jews and Arabs differ in their language, religion, and culture. In comparison to the Jewish population, the Arab population in Israel has lower socioeconomic status, is younger, is more likely to live in rural areas and has a lower life expectancy [12]. Since 1995, Israel has a National Health Insurance Law which provides health-care services for all Israeli residents regardless of religion, ethnicity, and gender. That is to say, Arabs and Jews are equally eligible to receive health services. They are treated in the same hospitals with the same health-care teams [13]. According to the Israeli Central Bureau of Statistics, the proportion of persons aged 65 and above in the entire population will increase from 10.6% at the end of 2013 to 14.6% at the end of 2035 [14, 15]. In the present study persons aged 65 and above are defined as elderly.

The objective of this study is to examine the risk of injury and health outcomes between ethnic groups, among hospitalized casualties, aged 65 and above.

## Methods

### Data resources

A retrospective study was performed which included all hospitalized casualties, aged 65 and above, who were recorded in the Israeli National Trauma Registry during a ten year period (1 January 2008 through 31 December 2017). During the study period up to 20 trauma centers (of 24 in Israel) participated in the Trauma Registry (all six Level Ι trauma centers and 14 Level ΙΙ trauma centers). All hospitals participating in the registry during the study period were included in the study. The Trauma Registry includes all injured trauma patients with an ICD-9-CM (International Classification of Diseases, Ninth Revision) diagnosis code of 800–959.9 who were hospitalized, including those who died in the emergency department, or discharged to another hospital following injury. The Trauma Registry does not include casualties who died at the scene of the event or on the way to the hospital, persons who were not hospitalized, or who were admitted 72 h or more after the event. All Jewish and Arab citizens of Israel, aged 65 and above, who were hospitalized due to an injury were included in this study. Foreign workers, tourists and unknown ethnicity were excluded (3%).

Data reported in the Registry is recorded by trauma registrars at each trauma center under the supervision of the trauma director and coordinator. Electronic files are transferred to the National Center for Trauma and Emergency Medicine Research, where quality assurance is carried out prior analyzing of data. The data in the Israel Trauma Registry is anonymous. The study received the approval of the ethical committee number 5138-18SMC.

### Measurements

The Registry includes demographic information, injury characteristics, hospital utilization, and disposition of each injured hospitalized patient. Ethnicity was categorized as Jews and Arabs. Injury Severity Score (ISS) was classified; 1–8 (minor), 9–14 (moderate), 16–24 (severe) and 25–75 (critical). Hospital length of stay (LOS) was categorized as < 7 and ≥ 7 days and admissions to the intensive care unit (ICU) were categorized as 0 and ≥ 1 days.

### Statistical analysis

SAS statistical software was used for data analysis and for comparison between the two ethnic groups. Statistical analysis included conventional tests such as χ^2^-tests and Fischer’s exact test for categorical data. The prevalence of injury hospitalization per 100 citizens was calculated among Jews and Arabs. Population size in each age group was based on the Israel Central Bureau of Statistics [15].

Multivariate logistic regression analysis was performed to calculate the odds of Jews relative to Arabs (odds ratios), undergoing surgical procedures, hospital LOS (cut-off ≥7), ICU stay, rehabilitation transfer and death; adjusted for age, gender, ISS and year of admission. Possible interactive effects were assessed for each model. A value of *p* < .05 was considered to be statistically significant.

## Results

### Prevalence of hospitalization due to injury

**Fig. 1:**
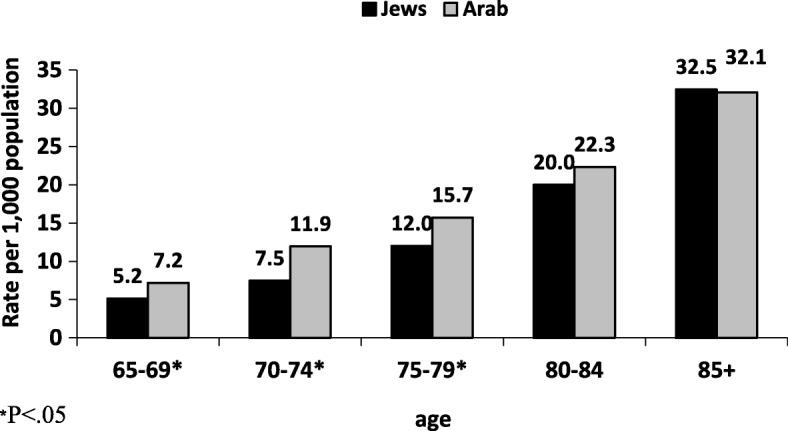
Prevalence of injury hospitalization per 1000 citizens, within each age group by ethnicity, in 2016

**Fig. 2:**
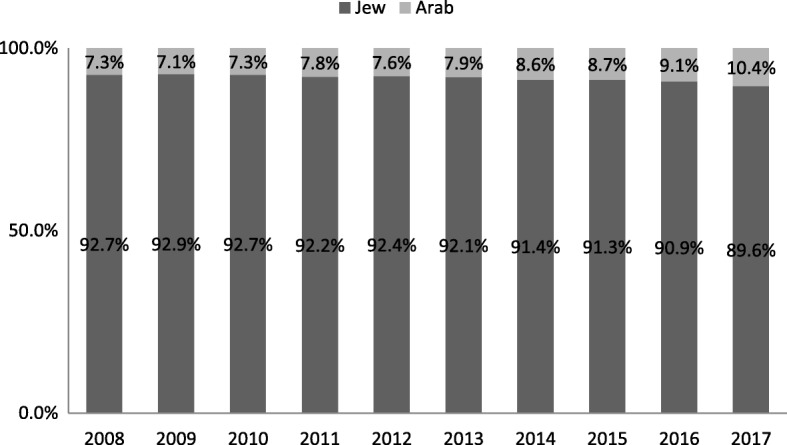
Hospitalized casualties aged 65+ by ethnicity, 2008–2017. Mantel-Haenszel chi-squre *p*.value<.0001

Between 2008 and 2017, 96,795 casualties, aged 65 and above, were hospitalized due to injury, representing 28.0% of all casualties in the registry (38.7% among Jews and 9.5% among Arabs). The overall prevalence of hospitalization due to injury among ages 65 and above was approximately 12.5 per 1000 citizens (based on the Central Bureau of Statistics, population distribution in 2016). Among Arabs the prevalence was greater (13.4/1000) than among Jews (12.4/1000) (Table 1). Since the age distribution between Jews and Arabs differs, we calculated the prevalence by age group for each population group (Table 2). In 2016, the prevalence of hospitalization due to injury for ages 65–79 was higher among Arabs compared to Jews, (*p* < .05) with no significant differences among the age group 80+ (Fig. 1). The proportion of hospitalized casualties aged 65 and above (31.0%) was 2.8 times greater than their proportion in the population (11.2%). The proportion of hospitalized elderly Arabs increased significantly during the study period, from 7.3% in 2008 to 10.4% in 2017 (Fig. 2).

**Table 1:** Population, number and percent of hospitalized casualties age 65+ by ethnicity, in 2016

	Total	Jews	Arabs
Population			
Total	8,546,000	6,768,500	1,775,000
Age 65+	958,600	879,100	79,600
Percent from the entire population	11.2%	13.0%	4.5%
Hospitalized casualties			
Total	38,546	27,434	11,112
Age 65+	11,967	10,897	1070
Percent from the entire hospitalized casualties^1^	31.0%	39.7%	9.6%
Rate per 1000 population^2^	12.5	12.4	13.4

^1^Calculation was based on: (Number of hospitalized casualyties Age 65+/ Number of hospitalized casualties) *100

^2^Calculation was based on: (Number of hospitalized casualties Age 65+/ Number of population age 65+) *1000

**Table 2:** Characteristics of hospitalized casualties aged 65+ by ethnicity, 2008–2017

	Total	Jews	Arab	*p*-value
Total	96,795 (100%)	88,836 (91.8%)	7959 (8.2%)	
Gender				
Female	63.7	64.4	55.9	<.0001
Age				
65–74	27.8	26.1	46.8	<.0001
75–84	40.6	41.0	36.8	
85+	31.6	32.9	16.4

### Population characteristics

Arab elderly represent 8.2% of elderly hospitalized casualties and 8.1% of severe/critical injuries. A higher percentage of hospitalizations were reported among Jewish women, in comparison to Arab women (64.4% vs. 55.9%, respectively, *p* < .0001). The Jewish population in Israel is older than the Arab population and similarly, the hospitalized casualties were older; casualties aged 75 and above constitute 73.9% of the Jewish casualties compared to 53.2% among Arab casualties (*p* < .0001). Jews, compared to Arabs, had a higher percentage of head injuries (10.5% vs. 8.9% respectively *p* < .001), but suffered from fewer lower extremity injuries, with the most significant difference being among the very old, aged 85+ (56.6% vs. 67.2% respectively for Jews and Arabs *p* < .001) (Table 3).

**Table 3:** Characteristics of hospitalized casualties aged 65+ by ethnicity and age group, 2008–2017

	Total		Ages 65–74		Ages 75–84		Ages 85+	
	Jew *N* = 88,836	Arab *N* = 7959	Jew *n* = 23,198	Arab *n* = 3725	Jew *n* = 36,374	Arab *n* = 2928	Jew *n* = 29,264	Arab *n* = 1306
Cause of injury								
Road accidents	9.2***	10.8	17.5***	14.7	8.7***	9.0	3.3	3.9
Falls	86.8	82.0	74.1	74.1	88.2	86.6	95.0	94.3
Other^a^	4.0	7.2	8.4	11.2	3.1	4.4	1.7	1.8
ISS								
Mild	36.5	36.8	47.7*	47.0	35.1***	30.2	29.6***	22.5
Moderate	54.0	53.9	41.9	43.7	55.2	59.2	62.0	71.0
Severe & Critical	9.5	9.3	10.4	9.3	9.7	10.6	8.4	6.5
Injured body region (AIS3+)								
Head	10.5***	8.9	10.4***	8.4	11.2	10.3	9.8**	7.3
Face	0.1	0.1	0.3	0.1	0.1	0.03	0.1	0.1
Thorax	3.8***	4.6	5.8	5.9	3.6	4.0	2.6	2.4
Abdomen	0.7	0.8	1.0	0.8	0.6*	1.0	0.4	0.3
Spine	0.9	0.8	1.3	1.0	0.9	0.7	0.7	0.6
Upper extremity	2.5**	2.0	4.1***	2.6	2.4*	1.6	1.4	1.1
Lower extremity	46.7*	48.0	32.2***	36.3	47.9***	54.4	56.6***	67.2
ICU (1+ days)	5.3***	6.2	6.0	5.9	5.5**	6.9	4.5	5.4
Undergone surgery	53.1***	55.0	50.3	49.7	53.0***	58.2	55.5	62.9
LOS (7+ days)	36.5***	29.4	29.9***	26.4	37.2***	32.5	40.8***	31.3
Rehabilitation	34.7***	18.0	24.7***	14.9	37.2***	20.6	39.4***	21.0
Inpatient mortality	3.2*	2.7	1.8	1.7	2.9*	3.6	4.5*	3.4

^a^Other include: burns, violence and unintentional injury

*ISS* Injury Severity Score, *AIS* Abbreviated injury score, *ICU* Intensive Care Unit, *LOS* Length of Stay in hospital

*p < .05 **p < .01 ***p < .001

### Fall injuries

Falls were the leading cause of injury among elderly casualties, constituting 86.8% of the hospitalizations among Jews and 82.0% among Arab casualties. Age stratified results showed that among elderly aged 75–84, a greater percentage of Jews were hospitalized with a fall injury in comparison to Arabs (88.2% vs 86.6% respectively) (Table 3). The majority of falls, 78.6% among Jews and 72.5% among Arabs, occurred on a level surface, *p* < .0001. While ground level falls were more frequent among Jews, falls from height were more frequent among Arabs compared to Jews (22.8% vs. 17.9%, respectively, *p* < .0001)**.** Among casualties hospitalized due to a fall, extremities were the most common part of the body injured, 70.7% of the casualties sustained extremities injuries, in both ethnic groups.

### Road traffic injuries

Road traffic injuries (RTI) constituted 9.2% of the hospitalizations among Jewish casualties compared to 10.8% among Arab casualties, *p* < .0001. Among elderly, aged 65–74, Jews were more likely than Arabs to be hospitalized due to a RTI (17.5% vs. 14.7% respectively for Jews and Arabs). In contrast, among elderly ages 75 and above the percentage of hospitalized Arabs due to a RTI was greater (Table 3). Among traffic related casualties, pedestrians were at greatest risk with Jews sustaining more pedestrian related injuries than Arabs (40.7% vs. 32.5%, *p* < .001). Among Arabs, the proportion of vehicle driver casualties was 1.6 times greater in comparison to Jews (34.8% vs. 21.5%, *p* < .001). Although Arab age 65 and above represent about 5% of the Arab population, among driver casualties they represent 14.6%. Only among vehicle drivers Arabs casualties had more severe and critical injuries (ISS 16+) compared to Jews (24.8% vs. 19.5% respectively, *p* = 0.04).

### Utilization of resources and discharge

Among severe/critical casualties and among casualties with severe head injuries, Arabs were more likely than Jews to be transported to the hospital by private car (27.3% vs. 21.1% respectively *p* < .001; 30.5% vs. 23.3% respectively *p* < .001), Data not presented. Over half of casualties underwent surgery, in both ethnic groups. Jews, compared to Arabs, underwent more head surgeries (2.0% vs. 1.2% respectively, *p* < .0001). Multivariate regression analyses, adjusted to age, gender, ISS type of injury, type of trauma center and year of hospitalization, were used to explore the effect of ethnicity on hospitalization characteristics and inpatient mortality (Table 4). The odds of being admitted to the ICU and being hospitalized for more than a week was greater for Jewish elderly compared with Arab elderly (OR 1.3 95%CI 1.192–1.326) However, it should be noted that differences in ICU admissions and LOS were found only among casualties not diagnosed with a head injury. Among elderly suffering from a head injury, no differences in ICU admissions or LOS were found between the two ethnic groups. Jews were 2.4 more likely to be discharged to a rehabilitation center in comparison to Arabs (2.4 OR 95%CI 2.224–2.525). There was no significant difference between the two ethnic groups in undergoing overall surgical procedures and inpatient mortality.

**Table 4:** Odds Ratio (OR) of Jews relative to Arabs by hospitalization characteristics and inpatient mortality; 2008–2017

Model	dependent variable	OR^a^ (Jews/Arabs)	95% Confidence intervals	*p*-value
1	Surgery	0.95	0.896–1.007	.086
2	ICU	1.27	1.140–1.422	<.0001
3	LOS 7+	1.26	1.192–1.326	< .0001
4	Rehabilitation	2.37	2.224–2.525	< .0001
5	Mortality	0.99	0.854–1.149	.902

^a^Adjusted for age (continuous variable), gender, Injury severity score - 3 groups (1–8, 9–14, 16–75), type of injury, type of trauma center and year of hospitalization for all models

*ICU* Intensive Care Unit, *LOS* Length of Stay in hospital

## Discussion

The present study not only characterizes injuries and hospital resource utilization among elderly casualties during the last decade in Israel, but also focuses on the differences between Jews and Arabs. The results show that the proportion of hospitalized casualties aged 65 and above among all hospitalized casualties was 2.8 times greater than their proportion in the overall Israeli population. The data presented illustrates the similarities and differences between Jews and Arabs regarding injury related hospitalizations. No significant differences between the two ethnic groups were found with regard to mortality or undergoing surgery. However, Jews, in comparison to Arabs, were more likely to have longer hospital stays,, to be admitted to the ICU and to be discharged to a rehabilitation center. Our results are in partial discrepancy with other international findings which find that there are disparities in the risk of injury and health outcomes between ethnic groups [3, 5–8, 16].These disparities may be attributed to cultural, safety and behavioral differences [17].

It was found that the disparities in the risk of hospitalization due to injury between Jews and Arabs are age-dependent. The risk of injury related hospitalizations was greater among Arabs compared to Jews, up to age 79. Previous research, which studied the relationship between socio-economic status and injury in Israel, found that in every socioeconomic cluster, the risk of hospitalization due to injury is higher among Arabs compared to Jews, even after adjusting for age [18]. While the Arab elderly are much younger than the Jewish elderly, they are more disabled [19]. The percentage of Arab elderly who are disabled and need help with activities of daily living is twofold that of Jewish elderly (30% vs. 14%, respectively). In addition, about one-quarter of the Arab elderly living in the community are homebound, compared to 12% among Jewish elderly [19].

Hicks et al. [10] documented that disparities in survival after injury exist between white and black patients depending on their age group. Younger white patients have better outcomes after injury than younger black patients, while older black patients have better outcomes than older white patients. Commonly posited explanation for these age-dependent disparities is the insurance status, availability of Medicare, and consequently, better access to pre injury medical care, in the older population. However, in Israel the National Health Insurance Law provides health-care services for all Israeli residents regardless of religion, ethnicity, and gender. Therefore, Arabs and Jews are equally eligible to receive health services.

Although the proportion of Arabs among elderly drivers (65+) in Israel is only 4.5% [20], we found that they represent 14.6% of vehicle drivers hospitalized due to road accidents. In addition, one in three elderly Arab vehicle drivers who were hospitalized due to a RTI suffered severe/critical injuries. These findings can be attributed to low quality of road infrastructure in Arab villages and towns, inadequate safety behaviors, and unlawful driving. Not only is road infrastructure inferior in Arab towns, but Arabs are more likely to drive in older vehicles and refrain from using safety accessories, such as seatbelts. [21, 22].

In our study several differences between Jewish and Arab elderly casualties were reported. Most notably, Arabs, in comparison to Jews, were far less likely to be discharged to a rehabilitation center (OR = 2.4). Other previous studies support our results that ethnic minorities are less likely to utilize rehabilitation facilities compared to the ethnic majority [3, 5]. For example, in the United States [5], ethnic minority patients are less likely to be placed in rehabilitation than non- Hispanic white patients, even after accounting for insurance status, suggesting existence of systematic inequalities in access. In addition, our previous study which compared Jewish and Arab children showed that Jewish casualties were more likely to be discharged to rehabilitation facilities than Arabs [3]. This fact can be attributed to various causes; including accessibility, culture and language. Residents living in the Northern district of Israel and in Jerusalem were reported to be 3–4 times less likely to receive inpatient rehabilitation compared to those living in the Center district [23]. Fifty percent and 17% of the Arab elderly live in the North district and Jerusalem, respectively, while only 9% live in the Center district [15].

In addition, there are vast differences in family relations and composition; Arab nuclear families in Israel usually occupy several households in the same village, which are proximate to each other. Children and grandchildren abound, and are actively involved in the lives of their parents and grandparents [19]. Arabs in Israel rated their culture as more tolerant toward their elders in comparison to Jews and spend more time with them [24]. In the Arab nuclear family, it is both expected and customary for the elderly to live at home and be cared for by family members Language barriers may also contribute to the differences. While the native language of the Arab population is Arabic, Hebrew is the official language in Israel. Due to communication barriers, elderly Arabs may prefer home care rather than residing in a rehabilitation facility with few Arab speaking professionals.

Another difference is the fact that among Arabs, a relatively high proportion of severe/critical casualties were transported by private car. This could be explained by the lack of ambulance availability in many Arab communities [25]. Moreover, in some of the Arab villages there is no exact address and it is difficult to direct the ambulance driver to a specific location. In addition, even though ambulance costs are often paid by health insurance, ambulance fees may be a deterrent. All of these reasons could contribute to the lack of ambulance use among Arabs.

Health policy decision makers and educators should aim to reduce the gaps between the two ethnic groups with regard to accessibility and availability of rehabilitation and ambulance services, as well as increase awareness regarding the availability and utilization of these pre and post hospital services.

Possible explanations for the differences in length of stay between Jews and Arabs may include geographic location and comorbidity. That is, maybe the longer length of hospital stay is due to preexisting conditions rather than the injury itself. In addition, since Arabs live in the periphery, they often arrive first at a level II trauma center after which are transferred to a Level I Trauma Center. Thus, the LOS may not reflect the total time of hospitalization. However, it should be reminded that among casualties suffering from head injuries, no differences in LOS or ICU were found between Jew and Arabs.

Implementing an in-depth study to explore the reasons contributing to disparities in health care utilization between Jews and Arabs is recommended. The outcomes of such a study will provide policy makers with evidence based data to reduce inequality between population groups.

Minority ethnic groups in the United State have a higher risk of mortality after injury than majority group [8, 9]. For example, African American and Hispanic casualties had higher odds ratio of death (1.17 and 1.47, respectively) compared to whites even after adjustment for demographic and injury severity variables [9]. In contrast, the outcomes of our study showed no significant differences between ethnic groups in inpatient mortality. Non-American studies support our results that ethnicity is not an independent predictor of trauma mortality outcomes [3, 11].

The first limitation of our study is that it includes only hospitalized casualties, as those who were discharged following treatment in the emergency department and those who died at scene of the accident or on the way to the hospital are not included. A second limitation is the lack of data regarding socioeconomic status. However, according to Goldman et al. factors other than socioeconomic differences are the cause for injury disparities between Jews and Arabs. A third limitation is the lack of data regarding distance from the scene of the accident to the hospital [26]. However, the INTR includes method of evacuation and transfer to the hospital, thus providing information regarding prehospital medical treatments.

## Conclusions

The outcomes of this study show that injury among the elderly is a burden to the health system. Since differences in injury characteristics, hospital utilization and discharge exist between Jewish and Arab elderly in Israel, it is vital that prevention programs be developed and adapted to meet the needs of each population group. Policy makers should increase the awareness among the Arab community of the available medical and rehabilitation services for the elderly population. In addition, decision makers in public health should use these results to optimize the allocation of pre and post hospital emergency care.

## Abbreviations

ICD: International classification of diseases
ICU: Intensive care unit
ISS: Injury severity score
LOS: Length of stay
SMC: Sheba medical center
